# MAX controls meiotic entry in sexually undifferentiated germ cells

**DOI:** 10.1038/s41598-024-55506-7

**Published:** 2024-03-04

**Authors:** Ayumu Suzuki, Kousuke Uranishi, Masazumi Nishimoto, Yosuke Mizuno, Seiya Mizuno, Satoru Takahashi, Robert N. Eisenman, Akihiko Okuda

**Affiliations:** 1 Division of Biomedical Sciences, Research Center for Genomic Medicine, Saitama Medical University, 1397-1 Yamane, Hidaka, Saitama 350-1241 Japan; 2 Division of Morphological Science, Biomedical Research Center, Saitama Medical University, 38 Morohongo, Moroyama, Iruma-gun, Saitama 350-0495 Japan; 3 Laboratory Animal Resource Center, University of Tsukuba, 1-1-1 Tennodai, Tsukuba, Ibaraki 305-8575 Japan; 4 Division of Basic Sciences, Fred Hutchinson Cancer Research Center, Seattle, WA 98109 USA

**Keywords:** Germ cell, Meiosis, Gametogenesis, Sexual differentiation, MAX, PRC1, Cell biology, Developmental biology

## Abstract

Meiosis is a specialized type of cell division that occurs physiologically only in germ cells. We previously demonstrated that MYC-associated factor X (MAX) blocks the ectopic onset of meiosis in embryonic and germline stem cells in culture systems. Here, we investigated the *Max* gene’s role in mouse primordial germ cells. Although *Max* is generally ubiquitously expressed, we revealed that sexually undifferentiated male and female germ cells had abundant MAX protein because of their higher *Max* gene expression than somatic cells. Moreover, our data revealed that this high MAX protein level in female germ cells declined significantly around physiological meiotic onset. *Max* disruption in sexually undifferentiated germ cells led to ectopic and precocious expression of meiosis-related genes, including *Meiosin*, the gatekeeper of meiotic onset, in both male and female germ cells. However, *Max*-null male and female germ cells did not complete the entire meiotic process, but stalled during its early stages and were eventually eliminated by apoptosis. Additionally, our meta-analyses identified a regulatory region that supports the high *Max* expression in sexually undifferentiated male and female germ cells. These results indicate the strong connection between the *Max* gene and physiological onset of meiosis in vivo through dynamic alteration of its expression.

## Introduction

Germ cells are the only cell type that transmits genetic information to the next generation. Meiosis, a fundamental process of gametogenesis, is a specialized type of cell division that halves the ploidy of the genome^1–3^. While the molecular bases of most events that occur during meiosis are highly conserved among eukaryotes, the regulatory mechanisms that control its onset in mammalian germ cells are largely unknown because of their limited evolutionary conservation^2–6^. Dysregulation of meiotic genes in germ cells is closely linked to infertility and cancer^6–9^, underscoring the importance of elucidating the detailed molecular mechanisms that control meiotic onset in mammals.

During fetal development, primordial germ cells (PGCs) are generated from several epiblast cells located in the posterior/proximal corner at approximately embryonic day (E)6.25. They migrate to the genital ridge by around E10.5. Up to E11.5, germ cells remain sexually undifferentiated while testis- or ovary-specific gene expression can be observed in somatic cells^10^. Germ cells undergo sexual differentiation by E12.5, based on differentiation signals from the surrounding somatic cells. Female germ cells initiate meiosis around E13.5 with the expression of meiotic genes in response to retinoic acid (RA)^11,12^. In contrast, male germ cells do not initiate meiosis during the embryonic stage, mainly due to degradation of RA by CYP26B1 and translational repression of mRNAs from meiosis-related genes by NANOS2, one of the crucial male-specific factors that promote and suppress male- and female-specific pathways, respectively^13,14^. The *Stra8* (*stimulated by retinoic acid 8*) gene is a crucial regulator of meiotic onset in male and female germ cells^15–18^. Although the molecular mechanisms by which STRA8 sustains the early stages of meiosis have long been enigmatic, identification of MEIOSIN as a binding partner of STRA8 revealed that STRA8 functions as a transcriptional activator that activates numerous meiosis-related genes by interacting with MEIOSIN, including the *Stra8* and *Meiosin* genes themselves^19^. Meiosis-related genes may need to undergo certain epigenetic changes before being activated by the STRA8/MEIOSIN transcriptional complex so that the complex can perform its function effectively. However, any such epigenetic regulation remains elusive.

Although MYC-associated factor X (MAX) is widely known as an obligate partner of MYC, activating numerous genes involved in cell proliferation and metabolism^20–22^, it also heterodimerizes with MGA. This MGA-MAX dimer constitutes polycomb repressive complex (PRC)1.6, an atypical subtype of PRC1^23,24^. Previously, we demonstrated that PRC1.6 suppresses meiotic onset in mouse embryonic stem cells (ESCs) and germline stem cells (GSCs), which are derived from preimplantation embryos and spermatogonial stem cells, respectively^25^. We previously showed that knockout of the *Max* gene in ESCs is accompanied by cytological changes reminiscent of germ cells undergoing meiotic prophase I, with global derepression of meiosis-related genes such as *Sycp3* and *Stra8*^25–35^. These cytological changes are independent of the expression of *Blimp1*, the gene encoding a master regulator of primordial germ cells^36^, but dependent on *Stra8* expression, suggesting that the meiotic germ cell-like cytological changes observed in *Max*-null ESCs are not secondary consequences of ESC differentiation into germ cells, but stem from these cells’ intrinsic ability to initiate meiosis ectopically. Consequently, we hypothesized that MAX acts as a direct and crucial regulator for the initiation of meiosis. In this study, we addressed this hypothesis by investigating the role of the *Max* gene in mouse primordial germ cells.

## Results

### Meiotic onset is preceded by a prominent decline in *Max* expression in female PGCs

**Figure 1:**
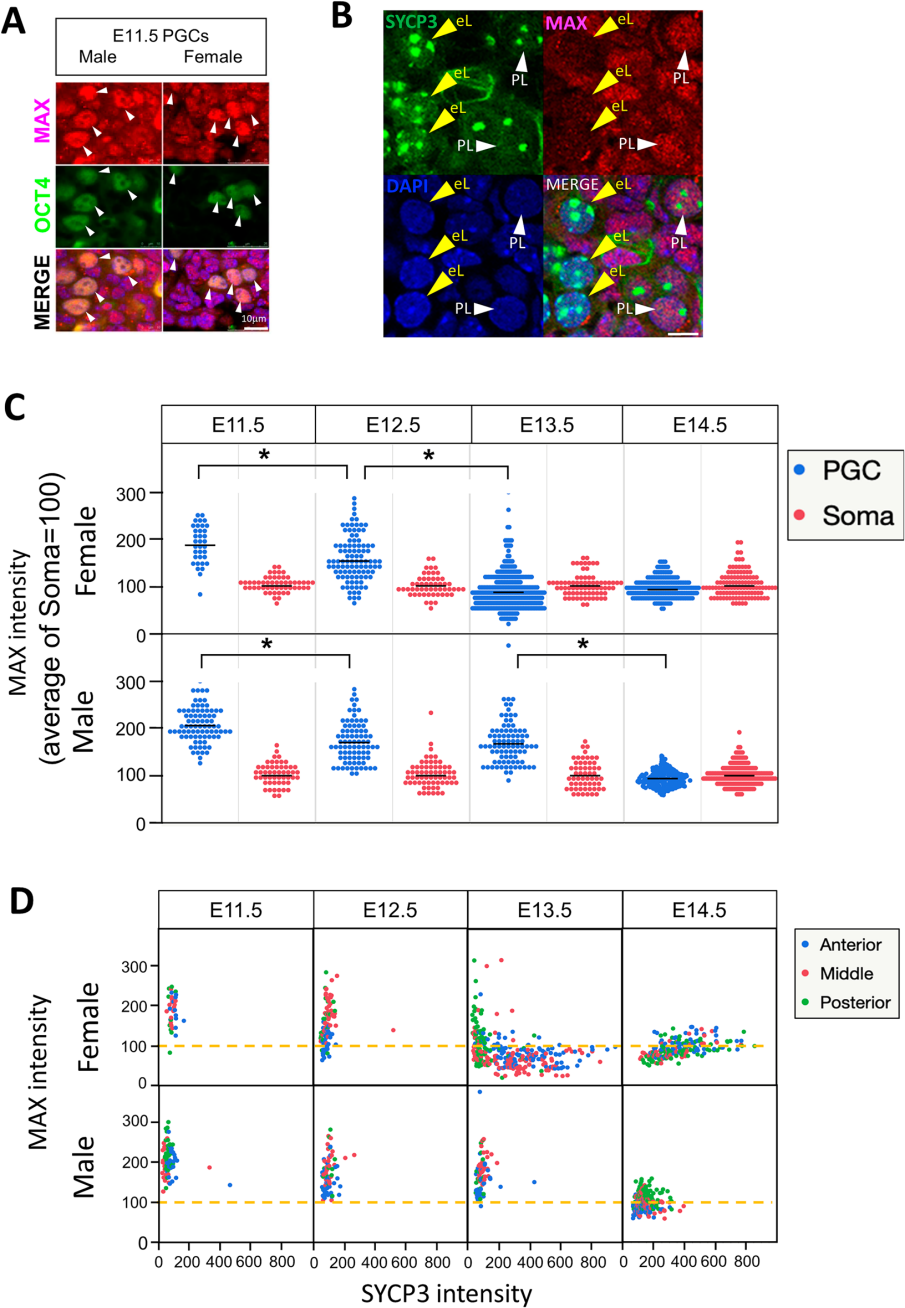
Spatiotemporal alterations of MAX protein levels in embryonic male and female germ cells. (**A**) Double immunofluorescence staining of MAX and OCT4 (POU5F1) in male and female gonads at E11.5. Arrowheads indicate each OCT4-positive germ cell. (**B**) Gonadal sections of female germ cells at E13.5 were immunostained for MAX and SYCP3, and counterstained with DAPI. PL, preleptotene; eL, early leptotene. (**C**) Plots of the intensity of individual MAX staining signals in male and female germ cells and somatic cells. The relative intensities of MAX staining in individual germ and somatic cells in male and female gonads at E11.5, E12.5, E13.5, and E14.5 were determined using ImageJ software. The mean MAX signal intensity in somatic cells was arbitrarily set to 100. (**D**) Scatter plots representing the intensities of the MAX and SYCP3 staining signals in individual male and female germ cells at E13.5 localized in the anterior (blue dot), middle (red dot), or posterior (green dot) of the gonads. The intensities of the MAX and SYCP3 staining signals in somatic cells were set to 100.

We previously demonstrated that MAX protein levels decrease notably prior to meiosis during spermatogenesis^25^, although *Max* is generally known to be expressed ubiquitously^22,37,38^. To further explore the correlation between *Max* expression and meiotic onset in germ cells in vivo, we examined MAX protein levels in male and female germ cells during the sexual differentiation process by immunohistochemistry. At E11.5, when PGCs were not sexually differentiated, OCT4-positive male and female germ cells had significantly stronger MAX protein staining signals than the surrounding OCT4-negative somatic cells (Fig. 1A). However, at E13.5, the MAX protein signal in female germ cells at the preleptotene stage—the transition stage shortly before entering meiotic prophase I^39^—became less intense than in E11.5 germ cells, and this less intense signal was prominently weakened further in the early leptotene stage (Fig. 1B). Quantification of the MAX protein levels during the mid-gestation stage revealed that the MAX protein signal was maintained mostly at a constant level in somatic cells in both sexes during the entire mid-gestation stage. However, female but not male germ cells showed a considerable decline in the MAX protein signal at E13.5, coinciding with the timing of their meiotic onset (Fig. 1C). Male germ cells also showed a significantly decreased MAX protein signal at E14.5, although the biological significance and cause of this reduction are unknown. Notably, while the lowest levels of the MAX protein staining signal in male germ cells at E14.5 were comparable to the levels in somatic cells, female germ cells at E13.5 had a lower level of the MAX staining signal than somatic cells. Analyses using publicly available transcriptome analysis data^40^ revealed that the most significant difference in *Max* expression levels between male and female PGCs was evident at E13.5 (Supplementary Fig. 1A), indicating that the difference in the *Max* mRNA levels between male and female germ cells during the mid-gestation stage is largely reflected by the MAX protein levels.

Meiosis occurs in female gonads along the anterior–posterior axis. It occurs restrictively in germ cells located at the anterior portion of the gonad at approximately E13.5, and then the region containing the germ cells undergoing meiosis expands and/or moves to the posterior region in a wave-like fashion^11,12,41^. Therefore, we used publicly available global gene expression data^42^ to examine whether expression levels of the *Max* gene, as well as pluripotency- and meiosis-related genes, were influenced by the location of the female germ cells in the gonad. Female germ cells located in the posterior part of the gonad at E13.5 had higher expression levels of *Max* and pluripotency genes (*Oct4* and *Nanog*), but lower expression levels of meiosis-related genes (*Dazl*, *Stra8*, and *Sycp3*), than germ cells located in the anterior portion (Supplementary Fig. 1B). Next, we examined the MAX and SYCP3 protein levels in male and female germ cells by double immunohistochemistry in relation to the spatiotemporal regulation of meiotic entry. Female germ cells in the posterior portion of the gonad also showed delays in the reduction and increase in MAX and SYCP3 protein levels, respectively, compared with those in the anterior portion (Fig. 1D, upper panels), strengthening the notion that the decline in MAX protein levels and meiosis are closely linked events. However, this inverse correlation between them was no longer evident at E14.5. In contrast to female germ cells, male germ cells, which do not produce SYCP3 protein at appreciable levels during the embryonic stages, maintained a mostly constant level of MAX protein until E13.5, followed by significantly decreased levels between E13.5 and E14.5. However, unlike female germ cells, there were no spatiotemporal differences (Fig. 1D, lower panels). Immunocytochemical analyses of female gonadal cells dissociated to single cells revealed the lowest MAX protein levels in the early leptotene stage. However, these levels elevated during the subsequent leptotene and zygotene stages (Supplementary Fig. 2). These results indicate that the decline in MAX protein levels in female germ cells is a transient event that occurs around their physiological meiotic onset.

### *Max* gene disruption is coupled to meiotic onset in sexually undifferentiated male and female PGCs

**Figure 2:**
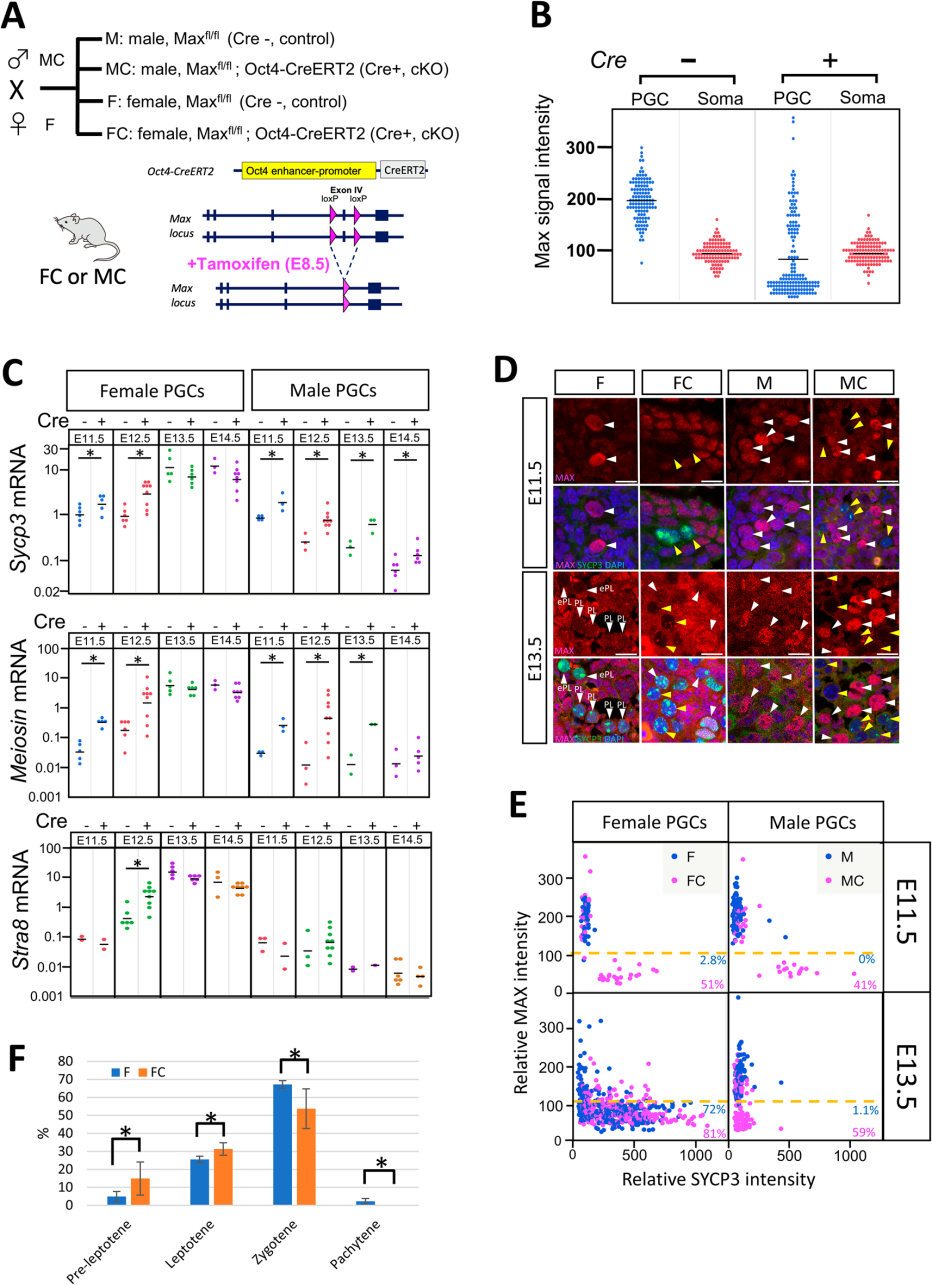
*Max* conditional knockout in sexually undifferentiated germ cells induces meiotic onset. (**A**) The mating scheme to generate *Max* conditional-knockout mice. Male and female *Max*^fl/fl^ embryos with CreERT2 cDNA were designated MC and FC, while male and female embryos without the cDNA were designated M and F, respectively. (**B**) Plots of the intensity of individual MAX staining signals in germ and somatic cells at E11.5 from tamoxifen-administered *Max*^fl/fl^ mouse embryos with and without the Cre transgene. MAX protein signal intensity was quantified using ImageJ software, as in Fig. 1C. (**C**) Examination of the effect of *Max* disruption on the expression of meiosis-related genes. *Sycp3*, *Meiosin*, and *Stra8* mRNA levels in germ cells from M, MC, F, and FC embryos at the indicated days were quantified by quantitative PCR. Data were normalized to the β-actin expression levels. A short vertical line within the dots represents the mean. Student’s t-tests were conducted, *P < 0.05. (**D**) Double immunostaining of germ cells subjected to *Max* gene disruption and their control counterparts. Fetal female and male embryos with or without CreERT2 cDNA were treated with tamoxifen at E8.5 and then sectioned genital ridges from embryos at either E11.5 or E13.5 were subjected to immunostaining of MAX (magenta) and SYCP3 (green), and counterstained with DAPI (blue). Each germ cell is indicated by an arrowhead. Yellow arrowheads indicate germ cells that completely lost the MAX protein signal. PL, preleptotene; eL, early leptotene. (**E**) Scatter plot representing the intensity of the MAX and SYCP3 staining signals in individual male and female germ cells at E11.5 and E13.5. Proportions of germ cells with a lower MAX protein signal than somatic cells are shown. (**F**) Proportions of germ cells in meiotic prophase l in gonads from F (n = 3305 total cells) and FC (n = 4456 total cells) embryos at E14.5. Germ cells were classified into four stages (preleptotene, leptotene, zygotene, and pachytene) by the SYCP3 staining pattern. The differences in the proportions between F and FC mice at each stage were examined statistically by Student’s t-test, *P < 0.05.

To further explore the relationship between *Max* expression and meiotic onset in germ cells, we conducted germ cell-specific knockout of the *Max* gene (Fig. 2A). To this end, we crossed *Max*^fl/fl^ female mice^43^ with *Max*^fl/fl^ male mice with the Oct4-CreERT2 transgene to produce MAX conditional-knockout (cKO) mice (*Max*^fl/fl^;Oct4-CreERT2 female and male mice, abbreviated as FC and MC, respectively), as well as control female and male *Max*^fl/fl^ mice (abbreviated as F and M, respectively). Both FC and MC mice harbor a homozygous *Max*-floxed allele (*Max*^fl/fl^ mice), wherein exon V can be excised through Cre activity, and a hemizygous Oct4dPE-CreERT2 transgene expressed under the control of a distal enhancer of the *Oct4* gene (Fig. 2A). To evaluate the efficiency of tamoxifen administration-mediated *Max* gene disruption, we injected tamoxifen into the abdomen at E8.5 and then performed immunostaining for MAX protein at appropriate embryonic stages. At E11.5, while Cre-negative control germ cells maintained a high MAX protein signal that was approximately twice as strong as that in somatic cells irrespective of tamoxifen treatment, most Cre-positive germ cells decreased the signal to a background level (Fig. 2B, see also germ cells indicated by a yellow arrowhead in Fig. 2D). We next examined alterations in the expression levels of meiosis-related genes (*Sycp3*, *Meiosin*, and *Stra8*) due to *Max* gene disruption in male and female germ cells by tamoxifen administration (Fig. 2C). Significant activation of the *Sycp3* and *Meiosin* genes in male germ cells was evident between E11.5 and E14.5 after the induction of *Max* gene disruption by tamoxifen administration. This activation was most prominent at E11.5 and E12.5, and the extent of the activation became less significant at E13.5 and E14.5, with the difference of *Meiosin* expression at E14.5 not being statistically significant. Female germ cells also showed the activation of these genes following *Max* gene disruption. However, such activation was evident only before sexual differentiation (E11.5 and E12.5). Indeed, expression of these genes in female germ cells subjected to Cre-mediated *Max* gene disruption was comparable to the level in controls without CreERT2 cDNA at E13.5, and lower at E14.5 (Fig. 2C). As for *Stra8* expression, our data revealed that the effect of *Max* gene disruption was distinct from that on *Sycp3* and *Meiosin* expression in which *Max* expression ablation did not notably affect *Stra8* expression levels in both male and female germ cells, except for at one particular time point, namely, E12.5, in female germ cells. We will discuss the regulation of *Stra8* expression of germ cells in the *Max*-null background, including its activation in female germ cells at E12.5, in more detail below (see Discussion). To further confirm that *Max* gene disruption in sexually undifferentiated germ cells leads to de-repression of other meiosis-related genes, we also compared expression levels of *Taf7l* and *Ddx4* between male and female *Max*-null germ cells at E11.5 and their respective control germ cells (Supplementary Fig. 3). These analyses revealed that, similar to *Sycp3* and *Meiosin* genes, the expression levels of both *Taf7l* and *Ddx4* were significantly elevated upon *Max* gene disruption, although the difference in *Ddx4* expression level in female germ cells was not statistically significant between tamoxifen-administered germ cells and their untreated controls.

Double immunohistochemical staining of MAX and SYCP3 confirmed the high level of MAX and little or no SYCP3 signal in the nuclei of sexually undifferentiated germ cells of control *Max*^fl/fl^ mice (F and M) at E11.5 (Fig. 2D,E). More importantly, a marked reduction in MAX signal by tamoxifen administration-mediated *Max* gene disruption in *Max*^fl/fl^ mice with the CreERT2 transgene was accompanied in most cases by the acquisition of a significant SYCP3 signal in male germ cells, and an even stronger SYPC3 signal in female germ cells, at E11.5 (Fig. 2D,E). Cytologically, these SYCP3-positive *Max*-null germ cells at E11.5 resembled germ cells at the preleptotene stage because of their clumped SYCP3 signal (Fig. 2D). However, this significant SYCP3 protein signal became hardly detectable in *Max*-ablated male PGCs at E13.5 (Fig. 2D,E). These results indicate that *Max*-ablated male germ cells did not maintain the meiosis-like cytological changes observed before sexual differentiation because of the reduction in the expression of meiosis-related genes during the meiotic process. Unlike male germ cells, female germ cells subjected to *Max* gene disruption retained the SYCP3 signal even at E13.5 (Fig. 2D,E). However, the immunostaining pattern of SYCP3 in germ cells at E14.5 revealed that germ cells at earlier (preleptotene and leptotene) and more advanced (zygotene and pachytene) stages were enriched and decreased, respectively, among germ cells subjected to *Max* gene disruption compared with the findings in control female germ cells that initiated meiosis physiologically (Fig. 2F). Taken together, these findings suggest that complete ablation of *Max* expression is sufficient to force the onset of meiosis in sexually undifferentiated male and female PGCs. However, such *Max*-null germ cells failed to undergo subsequent meiotic processes progressively and stalled immediately after the forced induction of meiotic onset. Next, we examined the effect of the ablation of *Max* expression on migrating PGCs. The SYCP3 signal intensity was extremely low in most MAX-null migrating germ cells at E10.5 (Supplementary Fig. S4A). However, some *Max*-ablated germ cells that had migrated to the vicinity of the genital ridge showed a clear SYCP3 protein signal (Supplementary Fig. 4A,B). These results indicate that the germ cells had acquired competence to respond to forced meiotic induction by *Max* ablation during their migration.

Next, we examined whether *Max* ablation in germ cells would affect the expression of genes related to sexual differentiation in the surrounding somatic cells as a secondary consequence of forced meiotic induction in germ cells. However, quantitative PCR analyses revealed that *Sox9*, which is crucially involved in male-specific characteristics^44,45^, did not have noticeably altered expression in either male or female gonadal cells (Supplementary Fig. 5A). Similarly, *Foxl2*, which plays indispensable roles in oocyte development and meiotic progression during the embryonic stage^46–48^, did not have appreciably altered expression (Supplementary Fig. 5B).

### Global expression analyses of the effects of *Max* gene disruption on embryonic germ cells

**Figure 3:**
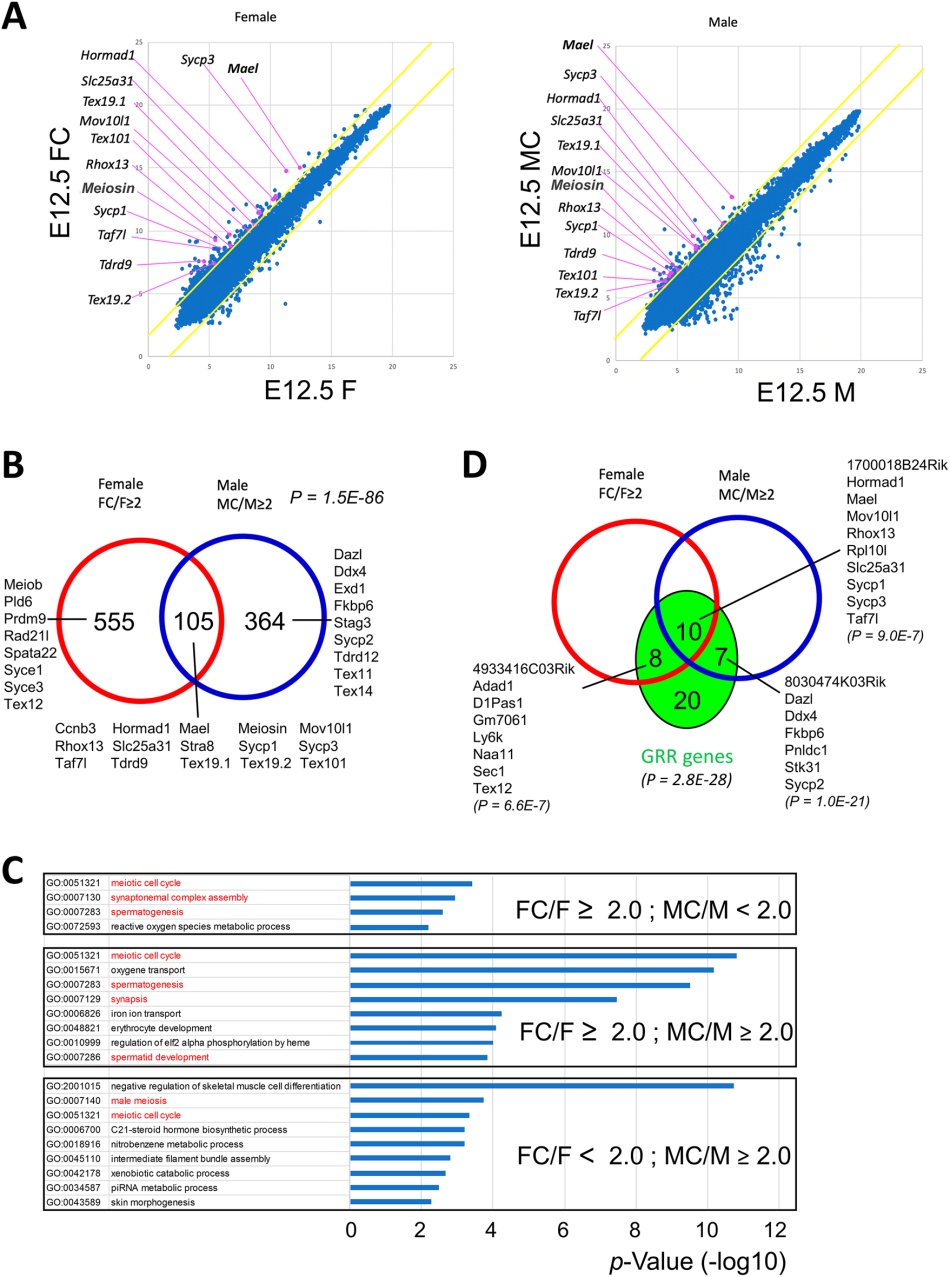
Alterations in the global expression profile of germ cells due to *Max* gene disruption. (**A**) Scatter plot of DNA microarray data from germ cells subjected to Cre-mediated *Max* gene knockout and their control counterparts without CreERT2 cDNA. The left and right panels show data from female and male germ cells at E12.5, respectively. Numerical values along the X- and Y-axes are the log_2_-transformed signal intensities of the microarray data. The upper and lower yellow diagonal lines in each panel indicate the boundaries of twofold up- and downregulation, respectively, due to *Max* gene disruption. Representative meiosis-related genes upregulated by *Max* disruption are individually marked by pink dots with gene symbols. (**B**) Venn diagram comparing genes activated more than twofold after Cre-mediated *Max* disruption in female and male germ cells at E12.5. The *P*-values for the significance of the overlap between two gene sets were calculated by hypergeometric tests. Meiosis-related genes included in each group are listed. (**C**) GO analyses of activated genes upregulated more than twofold after Cre-mediated *Max* disruption. Upregulated genes common to both male and female germ cells (middle panel) and those specific to either male (bottom panel) or female (upper panel) germ cells were subjected to GO analyses. Terms related to gametogenesis are indicated by red letters. (**D**) Venn diagram showing a significant overlap between genes activated by *Max* disruption in germ cells and genes designated as germline reprogramming-responsive genes^50^. *P-*values were calculated by hypergeometric tests, as in B.

To examine alterations in the gene expression profile caused by embryonic germ cell-specific *Max* gene disruption in an unbiased manner, we conducted DNA microarray analyses to compare global expression profiles between germ cells subjected to CRE recombinase-mediated *Max* gene disruption and their control counterparts lacking CreERT2 cDNA at E12.5 (Fig. 3A). The genes upregulated by *Max* gene disruption in female germ cells showed a statistically significant overlap with those in male germ cells, suggesting a shared role for MAX between male and female germ cells (Fig. 3B). Notably, a significant number of meiosis-related genes are included in all three gene sets; that is, genes commonly activated in male and female germ cells and those activated only in either male or female germ cells. Consistent with this, gene ontology (GO) analyses identified terms related to meiosis with all three gene sets, in which genes commonly activated in both sexes yielded those terms with higher statistical significance than in the two other gene sets (Fig. 3C). We also noted significant overlap between the genes upregulated by *Max* gene disruption and genes denoted as germline reprogramming-responsive (GRR) genes, whose transcriptional activation is closely linked to extensive epigenetic reprogramming in germ cells at E10.5–E11.5 via DNA demethylation of their high CpG promoters (Fig. 3D)^49^. This also supports the notion that control of the *Max* expression level is crucial for the initiation of meiosis because GRR genes play important roles in gamete generation and the initiation of meiosis^50^.

Although MAX was originally cloned as an obligate heterodimerization partner of MYC proteins, which are predominantly associated with the transcriptional activation of genes involved in cell proliferation and metabolic processes^20,21^, most genes defined as cell-type-independent targets of the MYC/MAX transcription complex^51^ did not show significantly altered expression (Supplementary Fig. 6). These results indicate that the major role of MAX at these developmental stages is not the promotion of cell proliferation and cellular metabolism by interacting with MYC.

### Germ cells with meiosis onset induced by *Max* ablation are eventually eliminated by apoptosis

**Figure 4:**
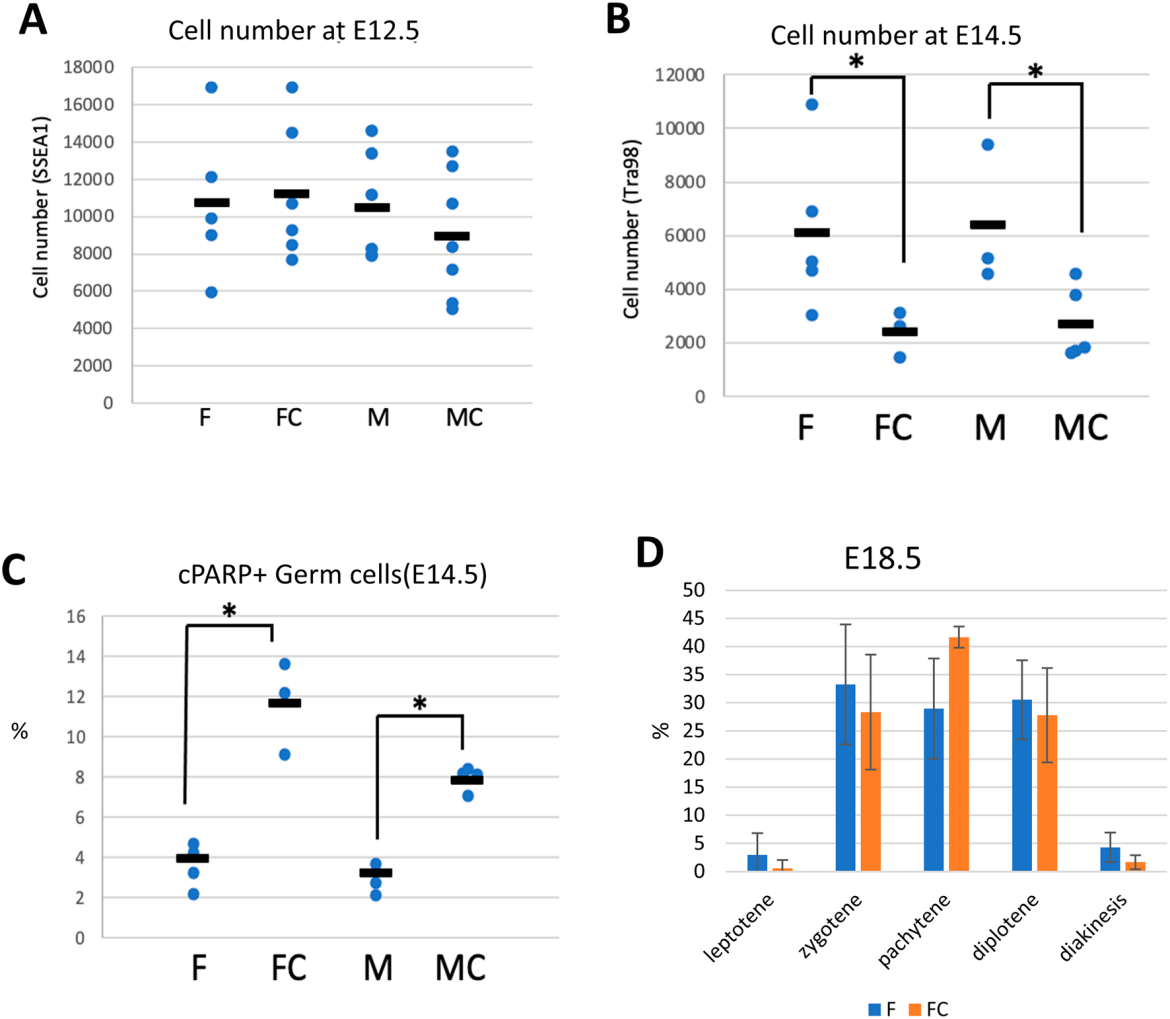
Apoptotic phenotype of primordial germ cells undergoing forced meiosis by *Max* ablation. (**A**, **B**) Total germ cell numbers assessed by TRA98 staining in gonads of M, MC, F, and FC mouse embryos at E12.5 (**A**) and E14.5 (**B**). (**C**) Percentages of cleaved-PARP-positive germ cells in gonads of M, MC, F, and FC mice at E14.5. (**D**) Proportions of germ cells in meiotic prophase l in E18.5 gonads of F and FC embryos. Germ cells were immunostained for SYCP3 after spreading onto a glass slide and their meiotic stages were classified as described in Fig. 2F.

Both male and female germ cells with forced meiosis onset induced by *Max* ablation stalled in the early stages of meiosis, and thus we determined the subsequent fate of these meiotically arrested germ cells. Although the total germ cell numbers in the genital ridges, assessed by the numbers of TRA98-positive cells, were unaffected by tamoxifen administration-mediated *Max* gene disruption at E12.5 in male and female embryos, a significant reduction in the germ cell number became evident in both sexes at E14.5 (Fig. 4A,B). To examine whether this reduction was due to apoptosis of *Max*-ablated germ cells, we performed immunostaining for cleaved PARP-1 as an indicator of the action of caspases^52,53^. In accordance with our hypothesis, there were significantly more cleaved-PARP-1-positive germ cells at E14.5 among the germ cells subjected to CRE recombination-mediated *Max* gene disruption than in controls without CreERT2 cDNA, for both males and females (Fig. 4C, Supplementary Fig. 7). Taken together, our data indicate that male and female germ cells that undergo artificial meiosis due to *Max* ablation are stalled at the early stages of meiosis, and are eventually eliminated by apoptotic cell death. Consistent with this idea, the skew of the distribution towards the earlier stages of meiosis at E14.5, observed in germ cells subjected to *Max* gene disruption (Fig. 2F), was no longer evident at E18.5 (Fig. 4D), indicating that the *Max*-null germ cells were mostly eliminated by apoptotic cell death before this stage, and that the vast majority of germ cells present at E18.5 are those that escaped CRE recombination-mediated *Max* gene disruption.

### Identification of the regulatory region that supports high *Max* expression in pluripotent embryonic stem cells and premeiotic germ cells

**Figure 5:**
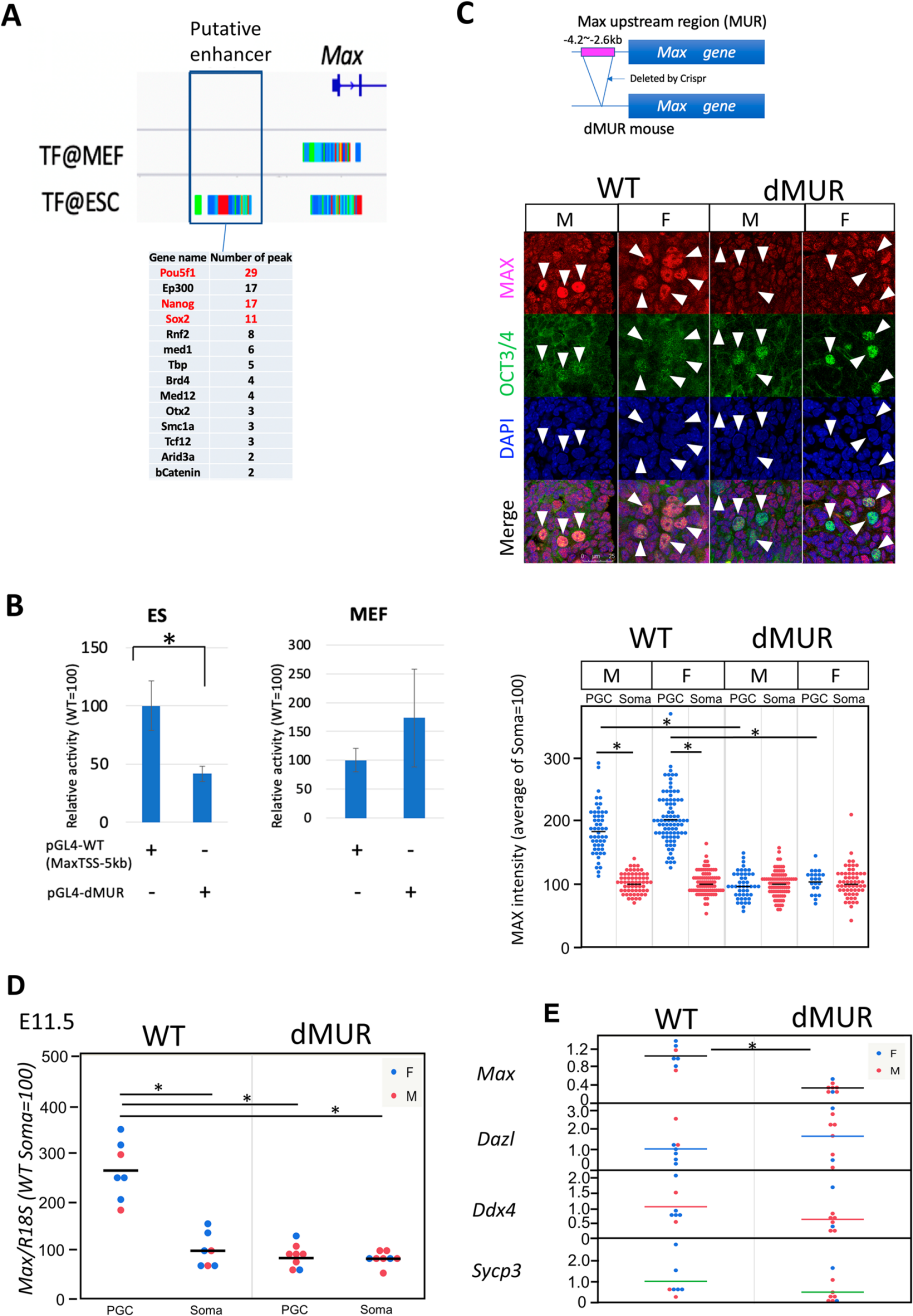
Identification of the positive regulatory region of the *Max* gene common to ESCs and premeiotic germ cells. (**A**) Analysis of ChIP-Atlas metadata identified a genomic region (MUR) that recruits representative pluripotent cell-specific transcription factors upstream of the *Max* gene. (**B**) Examination of the transcription-stimulating activity of MUR. Two luciferase reporters with or without MUR were individually introduced into mouse ESCs and embryonic fibroblasts by transfection, together with an internal control plasmid carrying the *Renilla reniformis* luciferase gene. A dual-Glo luciferase assay was conducted 48 h post-transfection. Data represent the mean ± standard deviation of three independent experiments. Student’s t-tests were conducted. *P*-values for the differences in luciferase activity due to the presence of MUR were 0.011 (marked by an asterisk) and 0.217 for ESCs and mouse embryonic fibroblasts, respectively. (**C**) Immunohistochemical analyses of MAX protein in germ cells of dMUR mutant mice. Upper panel illustrates the genetic manipulation of dMUR mice. Middle panel shows representative images of immunostained MAX and OCT4 in sections of genital ridges from wild-type and dMUR mice. Arrowheads indicate each OCT4-positive germ cell. The lower panel shows a plot of MAX protein signal intensity quantified by ImageJ software, as in Fig. 1C. The mean of the MAX signal intensity in somatic cells was arbitrarily set to 100. (**D**) Effect of MUR deletion on *Max* mRNA levels in germ cells. *Max* mRNA levels in germ and somatic cells of wild-type and dMUR mutant mice were quantified by quantitative PCR using *Rn18s* RNA level as an internal control. Blue and red dots indicate data from female and male samples, respectively. The asterisk indicates *P* < 0.05. (**E**) Effect of MUR deletion on meiosis-related gene expression in germ cells. Expression levels of *Dazl*, *Ddx4*, and *Sycp3* in germ cells of wild-type and dMUR mutant mice at E11.5 were quantified as in D. Expression levels of *Max* and meiosis-related genes in wild-type germ cells were arbitrarily set to 1. No statistically significant differences were evident in the expression levels between wild-type and dMUR mutant mice, except for in *Max* expression (*P* = 2.87 × 10^−6^; marked by an asterisk).

Several pluripotency marker genes that encode transcription factors, such as *Oct4* and *Nanog*, are also highly expressed in premeiotic germ cells and ESCs, as well as *Max*. Therefore, we hypothesized that *Max* contains a regulatory element that has a common function in germ cells and ESCs of recruiting commonly expressed pluripotency factors. To test this hypothesis, we searched for genomic regions in the vicinity of *Max* that are bound by certain pluripotency factors in mouse ESCs using ChIP-Atlas, a database of ChIP-seq data for meta-analysis (https://chip-atlas.org). As a result, we identified a genomic region that met these criteria (Fig. 5A). This genomic region, which was named MUR (*Max* Upstream Region), was located approximately 3 kb upstream of the transcription start site of *Max*. Analyses using publicly available DNase-sequencing data^54^ confirmed that MUR has an open chromatin configuration in ESCs and germ cells, but not in somatic cells (Supplementary Fig. 8A). We also investigated whether MUR bears characteristic histone modifications that generally characterize enhancers in ESCs and germ cells. Since no publicly available histone modification data of germ cells in vivo were available, we inspected data of germ cells generated from ESCs in vitro^55^. We found that mono-methylated histone H3 lysine 4 (H3K4me1) was more or less present around the region of MUR at all stages during the conversion of ESCs to primordial germ cell-like cells (PGCLCs). Meanwhile, levels of acetylated histone H3 lysine 27 (H3K27ac) dynamically changed during the conversion, in which rather high levels of H3K27ac in ESCs declined substantially in epiblast-like cells (EpiLCs) and such low H3K27ac levels persisted in d2PGCLCs, which are known to be most similar to in vivo germ cells at E7.0. However, the level of this modification was remarkably elevated in d4c7PGCLCs, which are transcriptionally similar to germ cells at E11.5. Since active and poised enhancers are generally known to be H3K4me1/H3K27ac-double-positive and H3K4me1-positive, but H3K27ac-negative, respectively^56^, these findings further support the hypothesis that MUR is involved in the transcriptional activation of the *Max* gene in ESCs and premeiotic germ cells as an active enhancer. To directly evaluate the transcription-stimulating activity of MUR in ESCs, we conducted luciferase assays using a luciferase reporter carrying MUR. The reporter with the MUR sequence induced two- or threefold higher luciferase activity than the reporter without MUR in ESCs, whereas no statistically significant difference in luciferase activity with MUR was evident in mouse embryonic fibroblasts (Fig. 5B). Next, we generated mice lacking MUR in their genome (dMUR) to assess the function of MUR in supporting *Max* expression in premeiotic germ cells. Although this deletion did not affect the germ cell number (Supplementary Fig. 8C), immunohistochemical analyses of MAX revealed that, compared with wild-type male and female germ cells, sexually undifferentiated germ cells lacking MUR showed a significantly lower MAX protein signal, comparable to that of surrounding somatic cells (Fig. 5C). This indicates that MUR contributes to the high *Max* expression in ESCs and premeiotic germ cells, which was supported by the quantification of *Max* mRNA (Fig. 5D). However, unlike complete ablation of *Max* expression, the decline in *Max* expression following MUR deletion was not accompanied by the induction of meiotic gene expression (Fig. 5E). These findings indicate that the decline in *Max* expression level to a level comparable to that of somatic cells is not sufficient to induce the onset of meiosis in germ cells, and thus these levels of *Max* gene expression need to decrease below those of somatic cells for the initiation of meiosis.

## Discussion

In this study, we demonstrated that conditional *Max* gene knockout enforces meiosis in both sexually undifferentiated female and male PGCs. However, these *Max*-null germ cells failed to progressively undergo subsequent processes. Indeed, *Max* ablation in female germ cells did not coordinate with the physiological meiotic onset at approximately E13.5 to further drive meiotic progression, but instead meiosis stalled, mainly around the leptotene-to-zygotene transition. Most *Max*-null male germ cells were positive for SYCP3 before sexual differentiation, but the high percentage of SYCP3-positive cells among these cells subsequently declined substantially, and SYCP3-positive cells were hardly detectable at E13.5. Our data also showed that male and female germ cells subjected to forced meiotic induction were eventually eliminated by apoptosis.

Although our data demonstrated that *Max* gene disruption was accompanied with de-repression of many meiosis-related genes such as *Meiosin* and *Sycp3* in sexually undifferentiated germ cells, that effect on the *Stra8* gene was completely different from that on other meiosis-related genes, indicating that, like in ESCs^35^, *Stra8* is not a direct target of PRC1.6. Our previous study also demonstrated that *Meiosin* expression in ESCs in a background of impaired PRC1.6 function sensitizes the *Stra8* gene to activation by RA^35^. Given that germ cells bear the same regulatory mechanisms as ESCs, it may be that removal of the PRC1.6-mediated repression of the *Meiosin* gene renders germ cells highly sensitive to *Stra8* activation by RA, which leads to the induction of meiotic onset. Therefore, we consider that the significant *Stra8* gene activation observed in *Max*-null female germ cells at E12.5 represents a secondary consequence of *Meiosin* gene activation, making germ cells susceptible to RA secreted from the mesonephros. Although the *Meiosin* gene was also activated in *Max*-null male germ cells, this was not accompanied by the activation of *Stra8*, probably because of the extensive degradation of RA by CYP26B1 produced in the Sertoli and testicular interstitial cells^18^.

This study also importantly demonstrates the dynamic changes of *Max* expression in germ cells, especially because *Max* expression levels are generally comparable across numerous cell types and are not strongly influenced by environmental changes^38,57^. Indeed, our data reveal that male and female germ cells maintain approximately twofold higher expression levels of *Max* than the surrounding somatic cells in a mitotically active state, but *Max* expression declines prominently at or immediately prior to meiotic onset in female germ cells. Because this decline is assumed to be linked to attenuation of the transcription-repressing activity of PRC1.6, it may be an important prerequisite for germ cells to undergo meiosis. It is not presently known how female germ cells reduce *Max* expression to a level lower than that in somatic cells. However, it seems likely that MUR—herein identified as a regulatory region that sustains high *Max* expression in both ESCs and mitotically active germ cells—substantially contributes to the subsequent downregulation of *Max* expression in female germ cells because the expression of genes encoding OCT4 and other pluripotency factors, which probably confer transcription-enhancing activity on MUR, shows a profound decline in female germ cells during this period. Our data also reveal that *Max* regains high expression around the zygotene stage in female germ cells, which may lead to the functional recovery of MAX-dependent activities, namely, MYC and PRC1.6 activities. In conjunction with the finding that *Max*-null male and female germ cells did not progressively undergo meiotic processes but were stalled, it seems likely that the recovery of MYC and/or PRC1.6 from their inactivated state via upregulation of *Max* expression is also required for germ cells to complete meiosis. Therefore, as the next step towards obtaining a complete understanding of the events occurring during the early stage of meiosis in germ cells at the molecular level, we will conduct future studies to elucidate how *Max* regains high expression after its low-expression state during meiotic processes, and how upregulation of *Max* contributes to the progression of meiosis.

## Methods

### Ethical statement

Animal experiments were carried out in strict accordance with international and institutional guidelines. The protocol was approved by the Institutional Review Board on the Ethics of Animal Experiments of Saitama Medical University (Permission numbers: 3136, 3137, and 3138). The study is reported in accordance with ARRIVE guidelines (https://arriveguidelines.org).

### Mice

Oct4dPE-CreERT2 transgenic mice were purchased from RIKEN BRC (BRC No. RBRC06509)^58,59^. Mice with a homozygous *Max*-floxed allele (*Max*^fl/fl^ mice)^43^ were bred with Oct4dPE-CreERT2 transgenic mice, and the resulting compound mice were bred with *Max*^fl/fl^ mice to obtain *Max*^fl/fl^;Oct4dPE-CreERT2^+^ mice. For tamoxifen administration, tamoxifen (Sigma-Aldrich, MO) was first dissolved in corn oil at a concentration of 10 mg/ml, and 0.5 mg (50 µl of the prepared solution) was injected intraperitoneally into pregnant mice at E8.5 days.

To generate mice lacking MUR, we used the guide RNAs 5′-CCCACTGAAACATCGCCCCATGG-3′ and 5′-TCTTCACAGGCTAAGATCTCAGG-3′. Unfertilized oocytes were collected from female C57BL/6 J mice (> 10 weeks old) superovulated with gonadotropin and chorionic gonadotropin and subjected to in vitro fertilization with sperm from C57BL/6 J mice. Five hours post-treatment, the resultant zygotes were subjected to guide RNA (5 ng/µl) introduction and GeneArt Platinum Cas9 Nuclease (100 ng/µl) (Thermo Fisher Scientific, MA) by electroporation using a NEPA 21 electroplater (NEPA GENE, Chiba, Japan), as described by Sato et al.^60^. Two-cell-stage embryos developed by subsequent in vitro culture were transferred into oviducts of pseudopregnant ICR female mice for development and delivery. One of the originally obtained heterozygous dMUR mutant mice was used as a founder to maintain the colony by crossing with wild-type C57BL/6 J mice. Heterozygous mutant mice were backcrossed three times before being used to generate homozygous mutant mice by heterozygous intercrosses. PCR using the following nucleotides was used to distinguish between wild-type and deletion mice:

Wild-type allele with the MUR sequence: forward, 5′-TTTTGGCTGGACTGGCTAAC-3′; reverse, 5ʹ-TTTTGGCTGGACTGGCTAAC-3ʹ.

Mutant allele without the MUR sequence: forward, 5ʹ-TTTTGGCTGGACTGGCTAAC-3ʹ; reverse, 5ʹ-TTTTGGCTGGACTGGCTAAC-3ʹ.

### Plasmid construction and luciferase assays

The 5ʹ flanking region of *Max* was amplified by PCR from genomic DNA of wild-type and mutant mice lacking MUR, using the following oligonucleotides: 5ʹ-GGTACCCCAGGAAGGATAGGCTGTGACG-3ʹ and 5ʹ-GTCGACGTAGTCCTCGAGCGTCGGAT-3ʹ. These PCR products were individually subcloned into the pGL4.10 vector carrying luciferase cDNA (Promega, WI). Plasmid transfection and subsequent luciferase assays were conducted as described previously^61^.

### Isolation of germ cells by MACS

To isolate germ cells by magnetic-activated cell sorting (MACS), gonads at E10.5–14.5 were dissected from embryos and dissociated to single cells using 0.05% trypsin–EDTA in PBS after removal of the mesonephros. After treatment with MACS buffer (2 mM EDTA and 0.5% BSA in PBS), germ cells were recovered using an anti-SSEA-1 antibody conjugated to MicroBeads (Miltenyi Biotec, 130-094-530), in accordance with the manufacturer’s instructions.

### Immunostaining

For immunohistochemical analyses, genital ridges isolated from embryos (E10.5–14.5) were fixed in 4% paraformaldehyde, embedded in OCT compound, and then frozen. Sections prepared from the frozen tissues were mounted on glass slides and subjected to immunostaining as described previously^25^. The primary antibodies used were as follows: anti-GENA (TRA98; BioAcademia, 73–003), anti-MAX (Santa Cruz Biotechnology, sc-197), anti-OCT4 (Santa Cruz Biotechnology, sc5279), and anti-cPARP (BD Pharmingen, 558,710). The anti-GENA antibody was used at 1:400 dilution and the other antibodies were used at 1:100 dilution. Immunostained tissues/cells were observed under a confocal laser scanning microscope (TCS SP8; Leica Microsystems, Wetzlar, Germany). The average staining intensities of MAX and SYCP3 in somatic cells were arbitrarily set to 100.

### Immunofluorescence staining for stage classification of germ cells in meiotic prophase

Preparation of germ cell nuclear spreads was conducted in accordance with the method described by Hwang et al.^62^ with some modifications. Briefly, after washing with PBS, gonads recovered from embryos were immersed in a low-tension buffer (17 mM trisodium citrate dihydrate citrate, 50 mM sucrose, 30 mM Tris–HCl pH 8.2, 1 mM EDTA, 0.5 mM DTT, and 0.1 mM PMSF) for 7 min. Each pair of gonads was suspended in a drop of 100 mM sucrose on a glass slide and gently dispersed by pipetting. The solution containing gonadal cells was then dropped onto slides soaked in a fixative solution (1% PFA and 0.15% Triton X-100) and pipetted to spread the cells. After repeated cell spreading, the glass slide was incubated overnight at room temperature in a humidified box and then dried at room temperature. The cells were then used in immunocytochemical analyses using antibodies against SYCP3 and MAX.

### Quantitative reverse-transcription PCR analysis

An RNeasy Micro Kit (QIAGEN) was used for total RNA preparation from germ cells isolated using MACS. The recovered RNAs were reverse-transcribed to cDNA using an FSQ-301 kit (Toyobo). TaqMan-based quantitative PCR was performed using the StepOnePlus Real-Time PCR System (Applied Biosystems, CA). All samples were normalized to the expression level of *Actb* (β-actin) or* Rn18s* (18S ribosomal RNA). The TaqMan probes used were as follows: *Max*, Mm00484802_g1; *Sycp3*, Mm00488519_m1; *Meiosin*, Mm01305445_m1; *Stra8,* Mm00486473_m1; *Dazl*, Mm03053726_s1; *Ddx4*, Mm00802445_m1; *Foxl2*, Mm00843544_s1; *Sox9*, Mm02619580_g1; *Actb*, Mm02619580_g1; and *Rn18s*, Mm03928990_g1.

### DNA microarray and GO analyses

Biotin-labeled cDNA was synthesized and then hybridized to Affymetrix GeneChip Mouse Genome 430 2.0 arrays (Affymetrix, CA), in accordance with the manufacturer’s instructions. Microarray expression data were background subtracted and normalized by the robust multiarray analysis method using R statistical software. Gene ontology (GO) analyses were conducted using DAVID web tools (http://david.abcc.ncifcrf.gov). The selected GO terms were subjected to further analyses using the AmiGO1 (http://amigo1.geneontology.org/cgi-bin/amigo/go.cgi) and REVIGO (http://revigo.irb.hr) websites to eliminate synonymous terms. The significance of the overlap between gene sets was assessed using the hypergeometric test.

### Supplementary Information

Supplementary Figures.

## Data Availability

Data from the DNA microarray analyses have been deposited in the NCBI Gene Expression Omnibus database under accession number GSE233556. Other data that support the findings of this study are available from the corresponding author upon request.
