# Supplementary material for: MAX controls meiotic entry in sexually undifferentiated germ cells
Source: Sci Rep. 2024 Mar 4;14:5236. doi: 10.1038/s41598-024-55506-7 (PMC10909893; doi:10.1038/s41598-024-55506-7)
Supplement: Supplementary file 1 — Supplementary Figures. [file 41598_2024_55506_MOESM1_ESM.pdf]

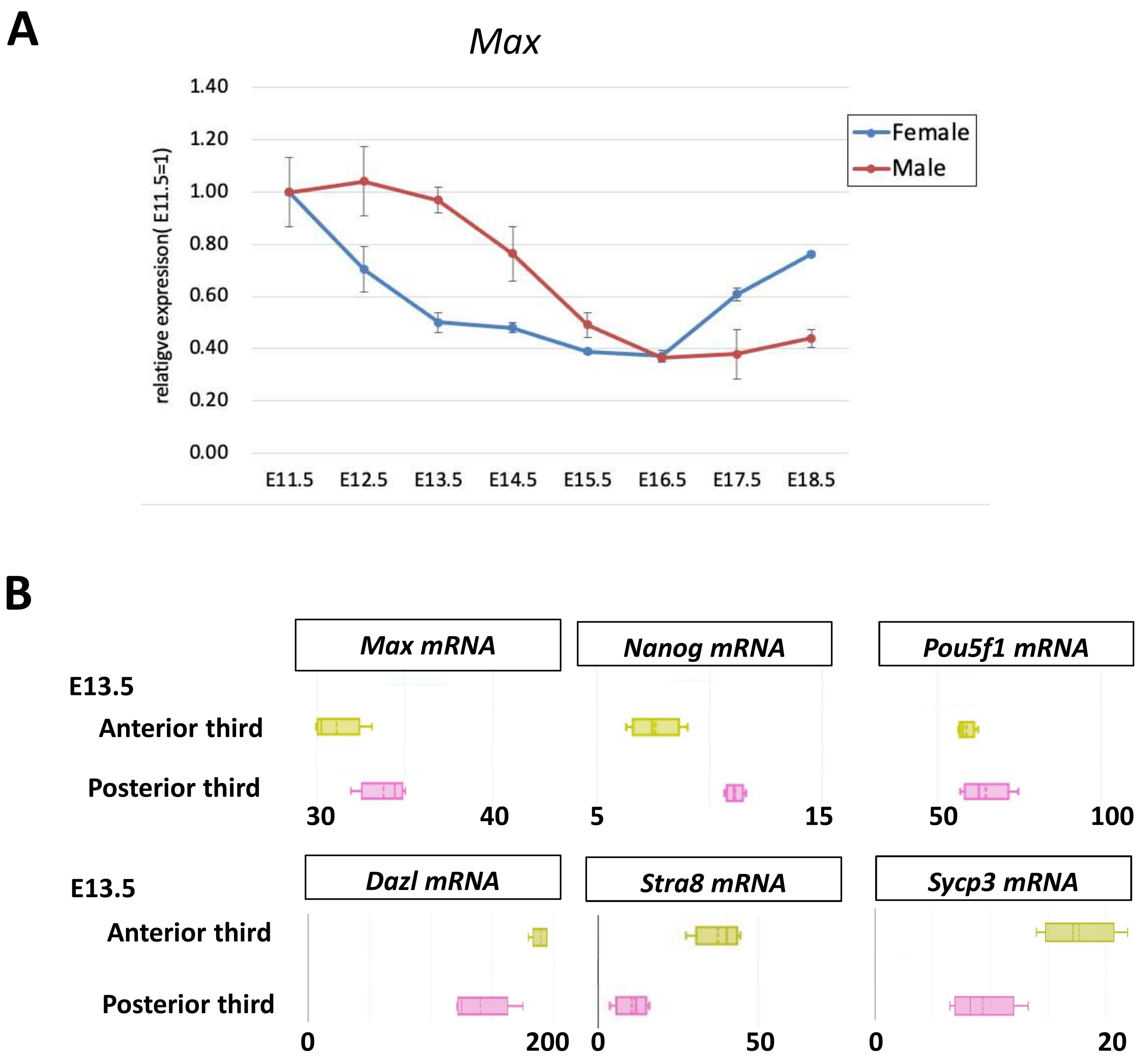

Supplementary Fig. 1. Spatiotemporal alteration of expression profiles of *Max* and pluripotency and meiosis-related genes in germ cells, related to Figure 1. (**A**) Expression dynamics of *Max* in male and female germ cells during the mid- and late-embryonic stages. Data were extracted from microarray data (GSE23322) published by Sabour et al.[40]. The *Max* expression levels in male and female germ cells at E11.5 were set to 1. (**B**) Expression levels of the *Max*, *Nanog*, *Oct4*, *Dazl*, *Stra8*, and *Sycp3* genes in the anterior and posterior portions of female gonads at E13.5. Box plots were generated using data reported by Soh et al.[42] via the ReproGenomics Viewer (https://rgv.genouest.org/).

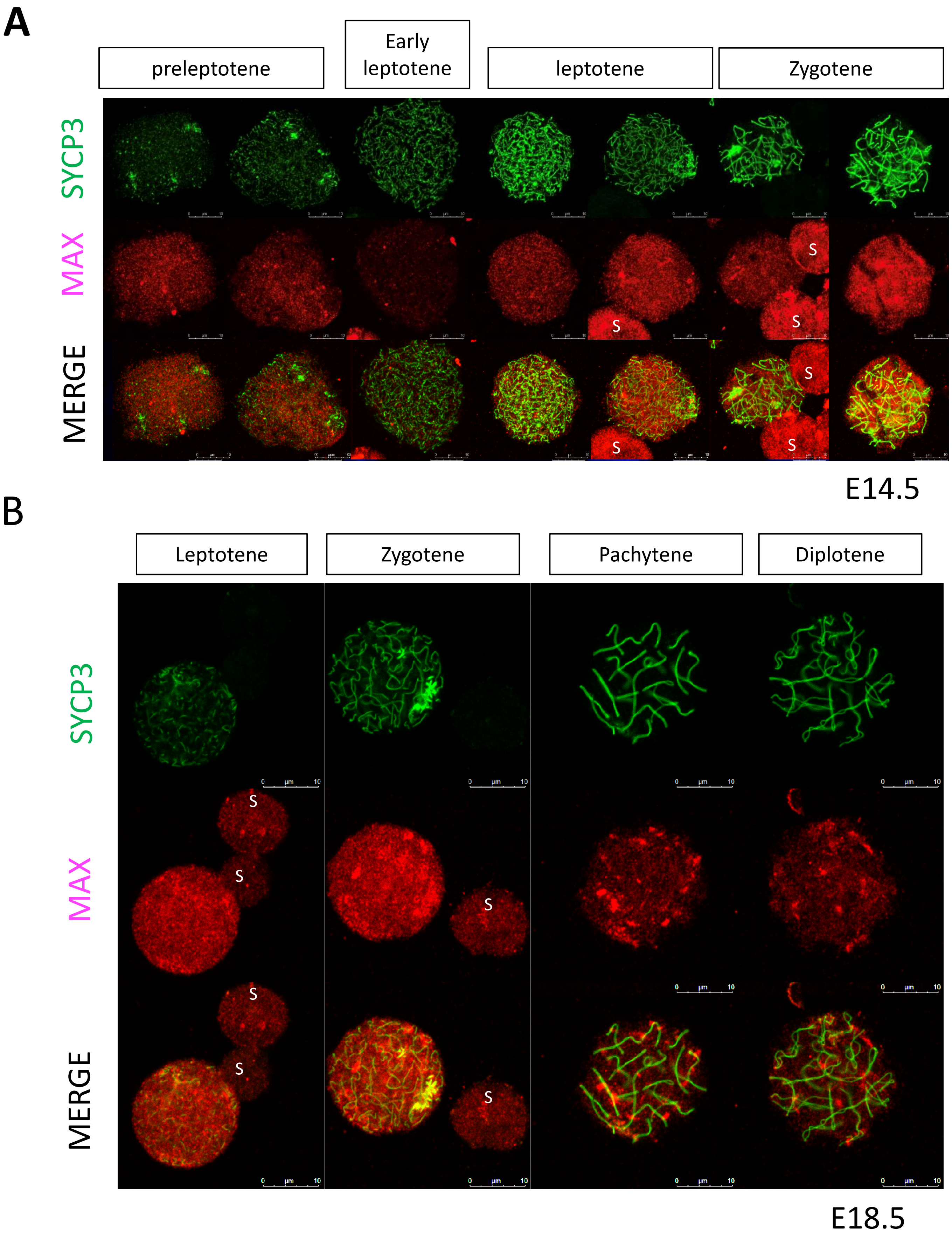

Supplementary Fig. 2. Immunocytochemical analyses of MAX and SYCP3 with female germ cells during meiotic prophase I, related to Figure 1. (**A**, **B**) Nuclear spreads of female germ cells at E14.5 (**A**) and E18.5 (**B**) were subjected to immunocytochemical analyses using anti-SYCP3 and anti-MAX antibodies. The stage of each germ cell was determined according to the SYCP3 staining pattern. S, somatic cells.

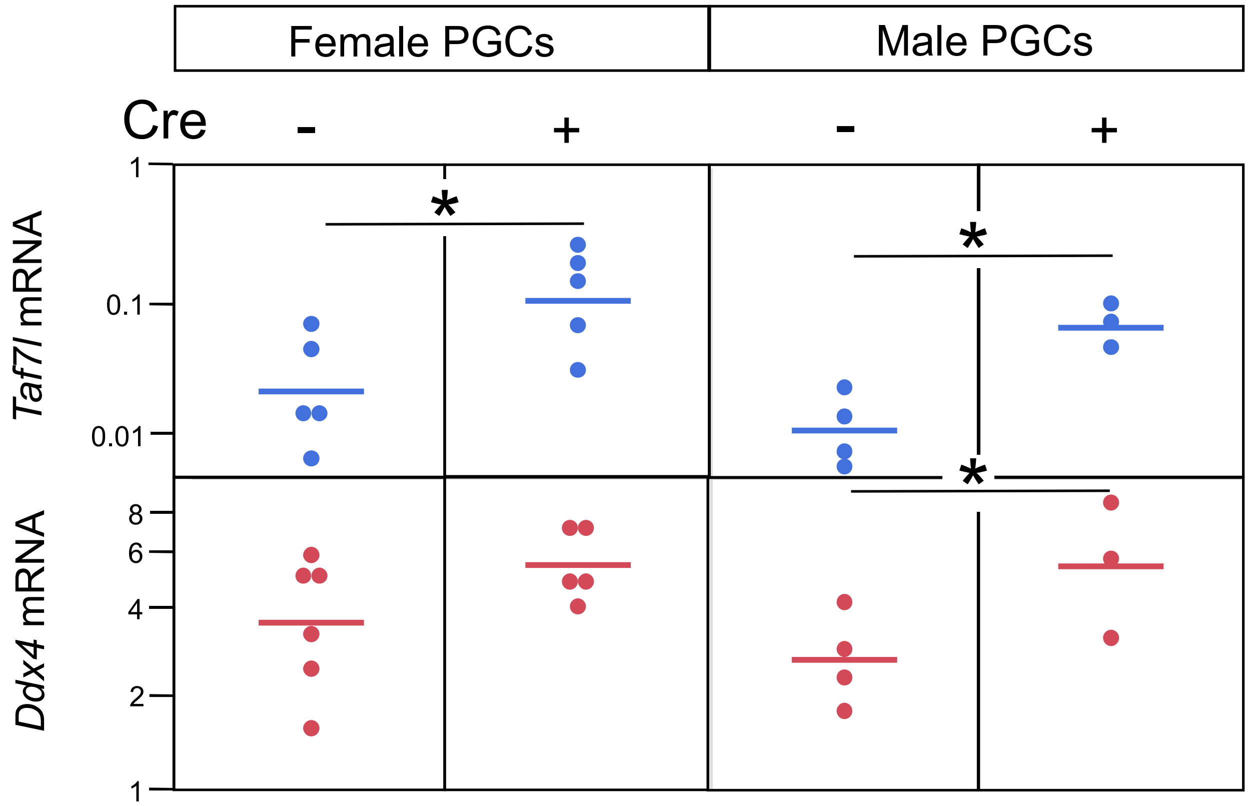

Supplementary Fig. 3. Examination of the effect of *Max* disruption on the expression of *Taf7l* and *Ddx4*. Quantitative PCR was conducted to quantify mRNA levels of *Taf7l* and *Ddx4* in germ cells from M, MC, F, and FC embryos at E11.5. Data were treated as in Figure 2C. Blue (*Taf7l*) and brown (*Ddx4*) vertical lines within the dots represent the mean. Student's t-tests were conducted, **P*<0.05.

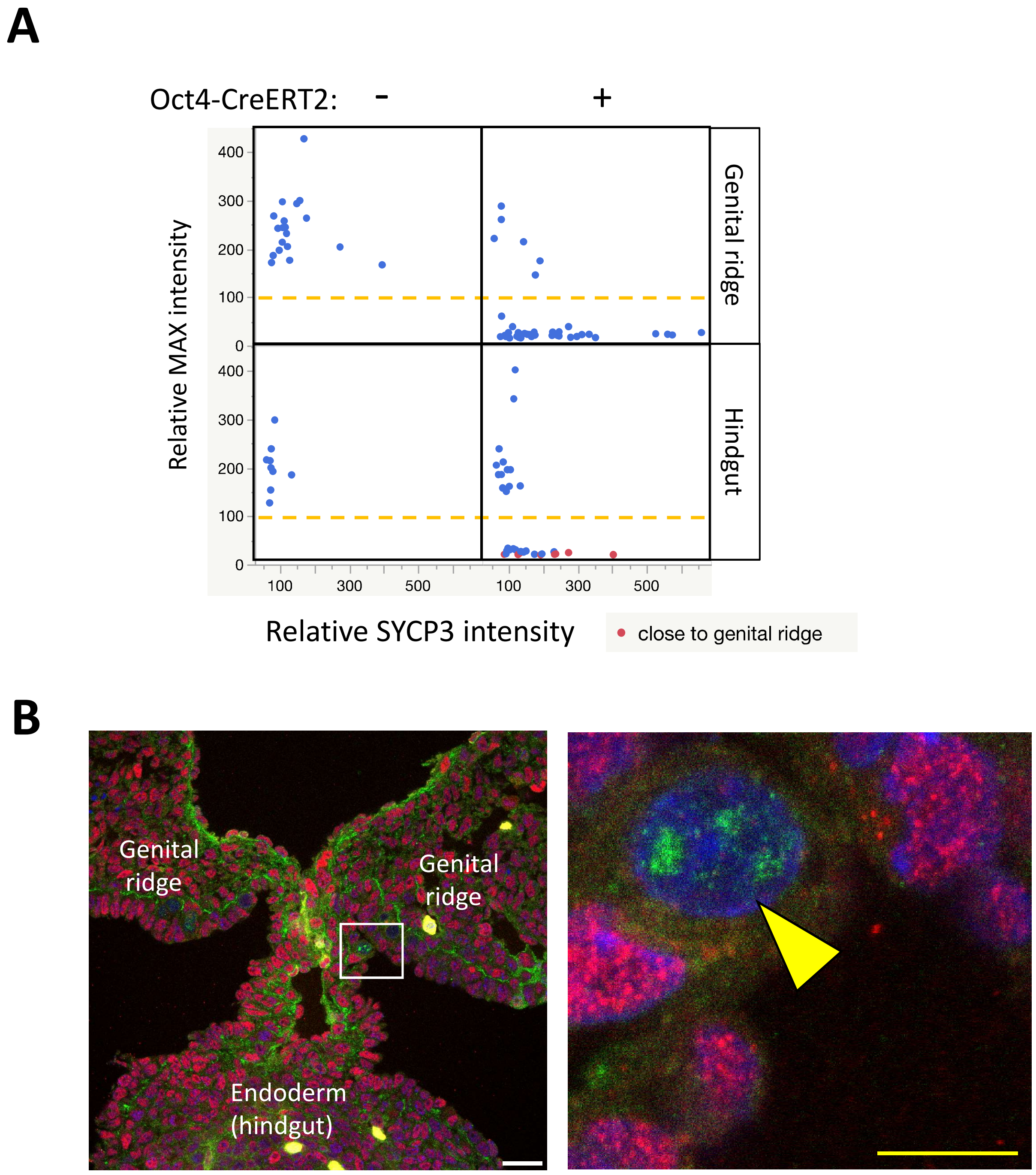

Supplementary Fig. 4. Effect of *Max* ablation on migrating PGCs at E10.5, related to Figure 2. (**A**) Scatter plot of the intensities of the MAX and SYCP3 staining signals in individual male and female germ cells at E10.5 in the genital ridge and hindgut that were either subjected to *Max* gene disruption by tamoxifen administration at E8.5 or control counterparts carrying no CreERT2 cDNA. The protein signal intensity was determined using ImageJ software, as in Figure 1C. The intensity of the MAX and SYCP3 signals obtained from somatic cells were set to 100. Data from germ cells that were located within the hindgut but close to the genital ridge are marked with pink dots. (**B**) Representative example of SYCP3-positive migrating germ cells that had migrated to the vicinity of the genital ridge. A region containing SYCP3-positive migrating germ cells is marked with a rectangle and its magnified image is shown on the right. White and yellow scale bars correspond to 25 and 8 μm, respectively.

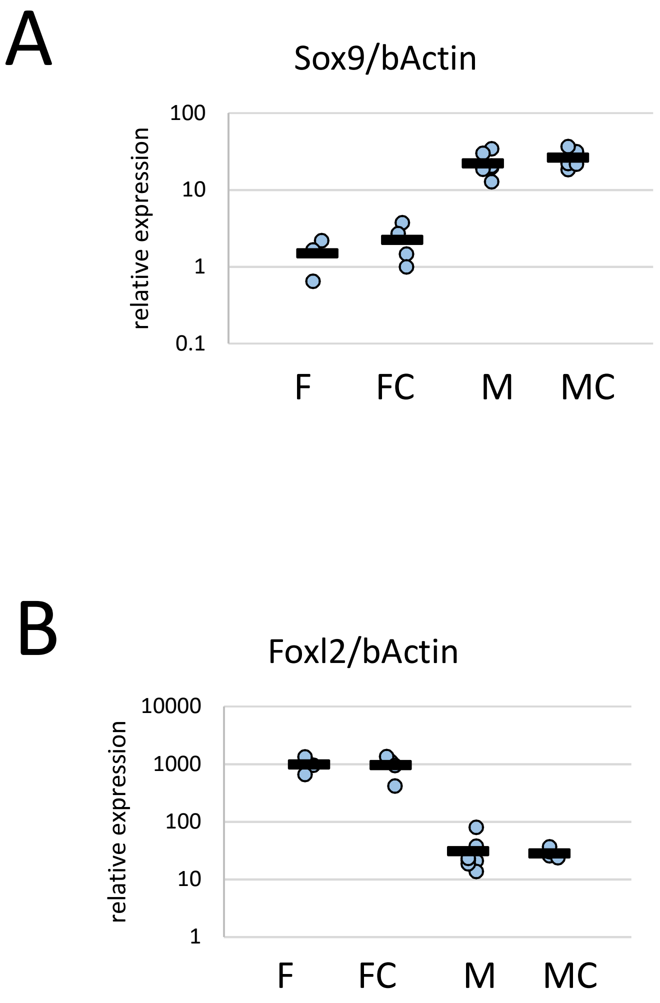

Supplementary Fig. 5. *Max* gene disruption was not accompanied by alterations to the expression of sex differentiation-related genes , related to Figure 2. (**A**, **B**) Expression levels of the *Sox9* (**A**) and *Foxl2* (**B**) genes were quantified by quantitative PCR with RNAs from whole gonads of E12.5 embryos that were subjected to Cre-mediated *Max* gene disruption by tamoxifen administration at E8.5 (FC and MC), or control counterparts carrying no CreERT2 cDNA (F and M).

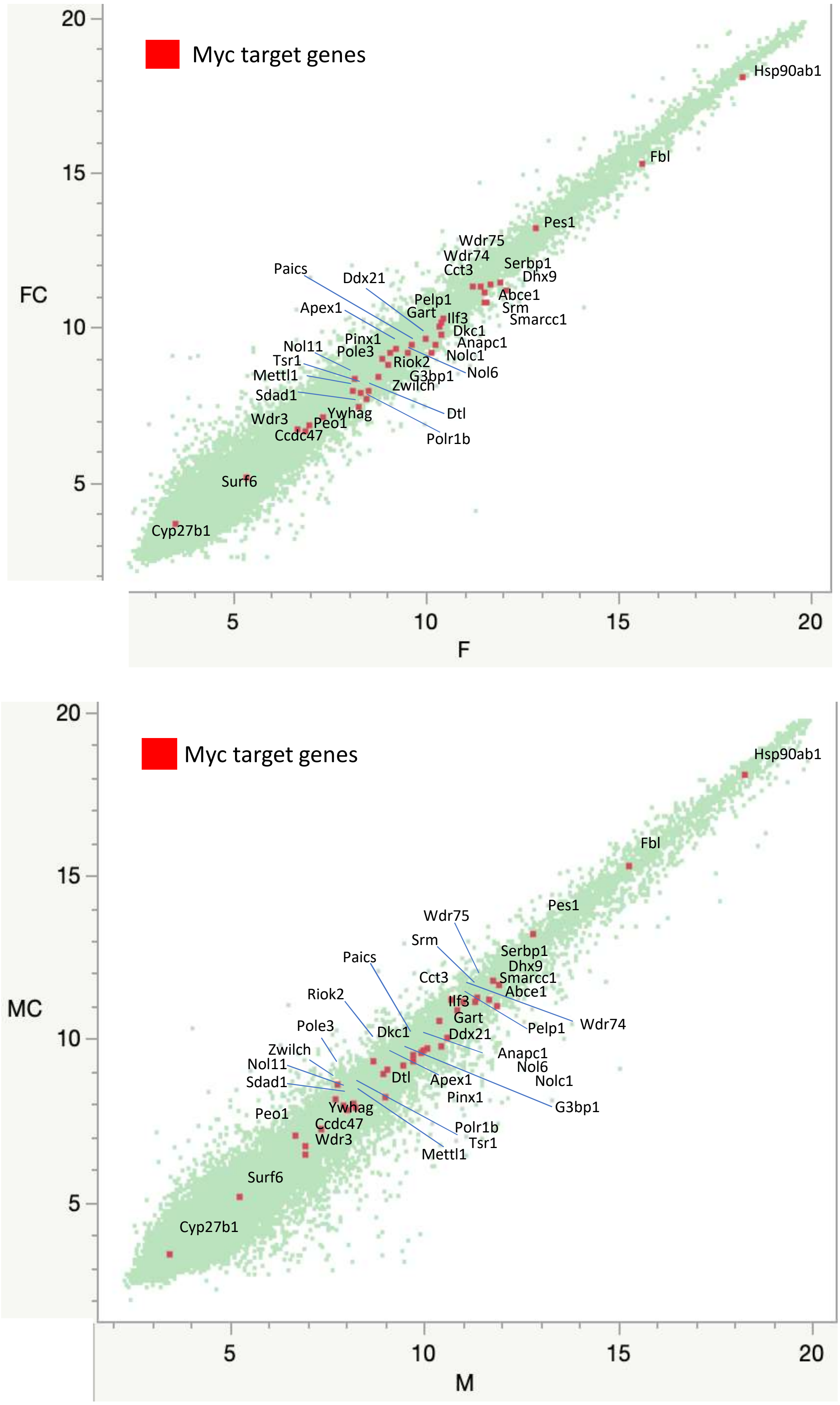

Supplementary Fig. 6. Effect of *Max* ablation on MYC target genes, related to Figure 3. Data of genes designated as cell-type independent core MYC targets[51] are marked with red dots with gene symbols on scatter plots of the DNA microarray data shown in Figure 3A. The upper and lower panels show data obtained with female and male germ cells, respectively.

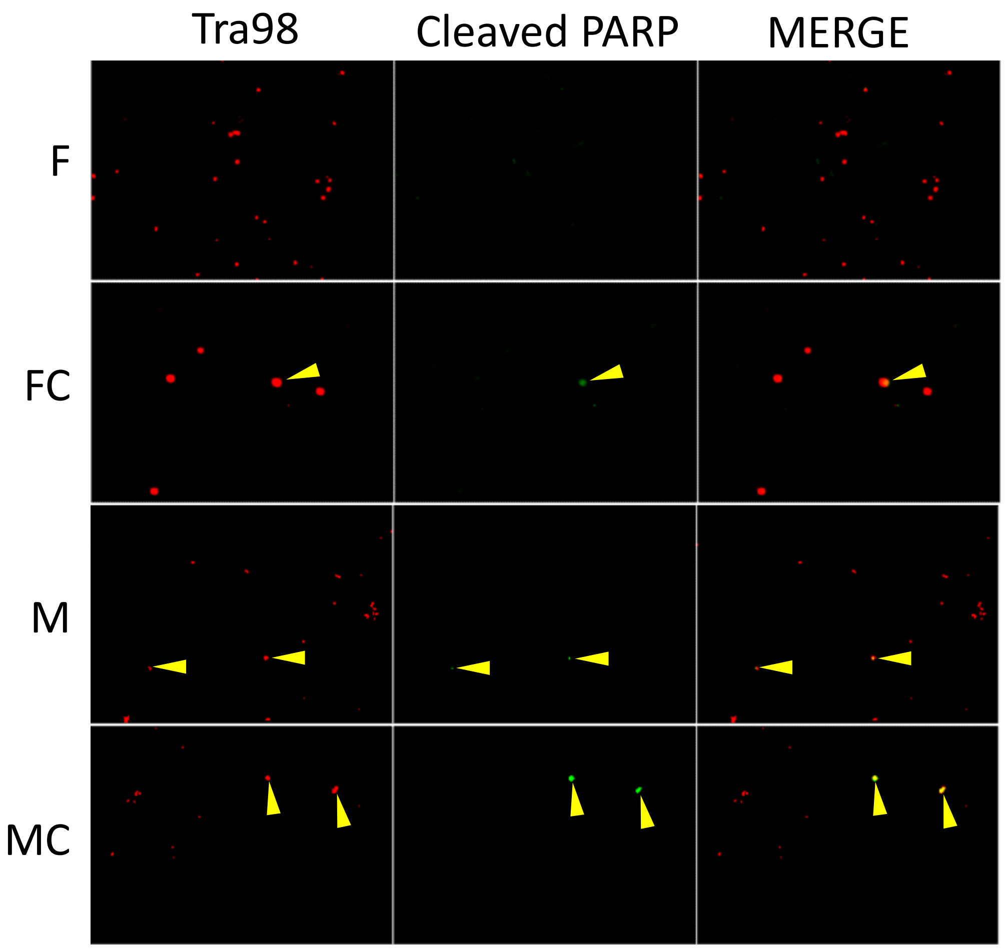

Supplementary Fig. 7. Germ cells forced artificially into meiosis by *Max* ablation showed an apoptotic phenotype , related to Figure 5. Representative images, double immunostained for TRA98 and cleaved PARP, of germ cells from whole gonads of embryos at E14.5 that were subjected to Cre-mediated *Max* gene disruption by tamoxifen administration at E8.5 (FC and MC), or control counterparts carrying no CreERT2 cDNA (F and M). TRA98/cleaved-PARP double-positive germ cells are indicated with yellow arrowheads. Percentages of cleaved PARP-positive positive germ cells in F, FC, M and MC are shown in Figure 5C.

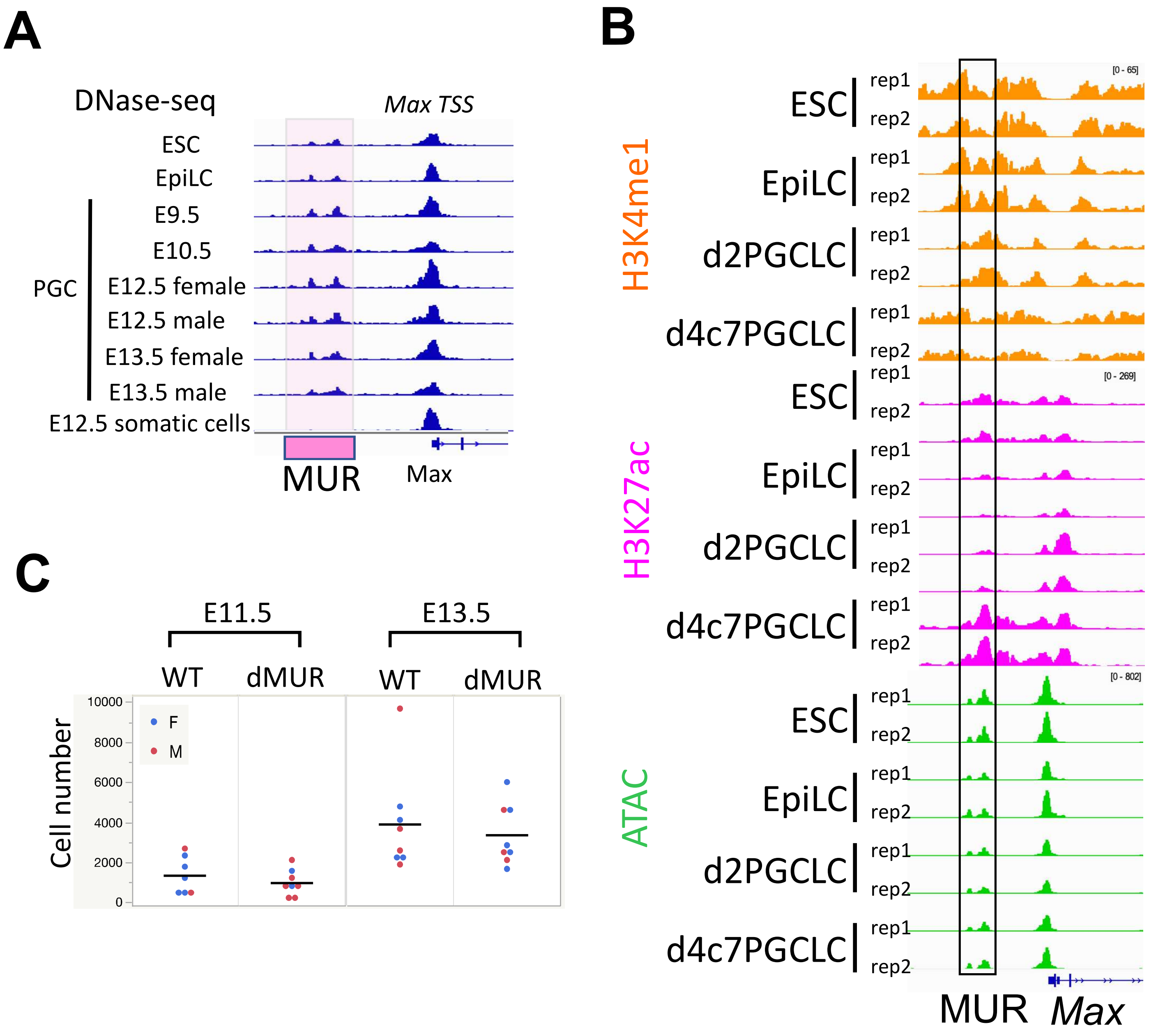

Supplementary Fig. 8. Chromatin accessibility and histone modification statuses of MUR in pluripotent cells and germ cells at the premeiotic stage and the consequence of its loss on total germ cell number, related to Figure 6. (**A**) DNase hypersensitivity of the region around the transcription start site of the *Max* gene and its 5′-flanking region. Publicly available data for ESCs (SRX3606939), EpiLCs (SRX3606943), and PGCs at E9.5 (SRX3606915), E10.5 (SRX3606917), E12.5 females (SRX3606919), E12.5 males (SRX3606923), E13.5 females (SRX3606925), E13.5 males (SRX3606927), and somatic cells at E12.5 (SRX3606921) reported by Li et al.[54] were used to generate this snapshot. (**B**) Histone modifications and ATAC-sequence data of the region around MUR and transcription start site of *Max* gene. Histone modification and ATAC sequence data of ESCs, EpiLC, d2PGCLC and d4c7PGCLC were obtained from functional genomic data depository of GEO under the accession numbers of GSE183826 and GSE183801, respectively. (**C**) Total germ cell numbers assessed by TRA98 staining in gonads of wild-type and mutant mice lacking MUR at E11.5 and E13.5. No statistical difference in total germ cell number between wild-type and dMUR-mutant mice was evident.
